# Sulfur Detection in Soil by Laser Induced Breakdown Spectroscopy Assisted by Multivariate Analysis

**DOI:** 10.3390/ma14030541

**Published:** 2021-01-23

**Authors:** Odhisea Gazeli, Dimitrios Stefas, Stelios Couris

**Affiliations:** 1 Department of Physics, University of Patras, 26504 Patras, Greece; gazeli.odhisea@ucy.ac.cy (O.G.); d.stefas@iceht.forth.gr (D.S.); 2 Institute of Chemical Engineering Sciences (ICE-HT), Foundation for Research and Technology-Hellas (FORTH), 26504 Patras, Greece

**Keywords:** laser-induced breakdown spectroscopy (LIBS), sulfur, soil, multivariate analysis

## Abstract

Laser-induced breakdown spectroscopy (LIBS) is used for the detection and determination of sulfur content in some organic soil samples. The most suitable sulfur spectral lines for such tasks were found to occur in the vacuum ultraviolet (VUV) spectral region and they were used for the construction of calibration curves. For the analysis, both univariate and multivariate statistical models were employed. The results obtained by the different analysis techniques are evaluated and compared. The present study demonstrates both the applicability and efficiency of LIBS for fast sulfur detection in soil matrices when aided by multivariate analysis methods improving the accuracy and extending the potential use of LIBS in such applications.

## 1. Introduction

The knowledge of the elemental composition of soil provides valuable information for the qualitative assessment of the different types of soil, allowing the better growth of plants and the higher quality of agricultural products. Fertilizers used in agriculture provide the essential metallic and nonmetallic elements (e.g., Al, Mg, Cu, Zn, Fe, C, P, etc.) for the growth of plants [1,2]. Among them, the addition of sulfur, which is of interest in the present study, can lead to higher performance of crops. However, unreasonable use of fertilizers can have impacts on public health, while it can result in environmental contamination issues as well. In particular, the presence of excess sulfur in soil may have negative effect on the crops’ growth. The qualitative/quantitative investigation of the soil content is an important parameter for several environmental studies. A typical example is the identification of various polluting substances [3]. For this purpose, the development of fast and efficient techniques capable of in situ and/or remote operation for the determination of metallic and non-metallic elements in soils is of high interest.

For the determination of soil’s metallic and/or nonmetallic content, different elemental analysis techniques have been proposed in the literature [4]. However, the requirement for fast and in situ analysis limits their applicability and, thus, only a few choices are realistically available. In addition, even if some of these techniques can provide accurate results, they have a high operational cost and they are time-consuming, therefore limiting their application for routine operation. In contrast to other techniques, laser-induced breakdown spectroscopy (LIBS) is a very popular and fast-developing technique. LIBS is based on the spectral analysis of the radiation emitted from a plasma that results from the interaction of an intense laser beam with a sample’s surface. Since every element exhibits some unique emission spectral lines, i.e., a kind of spectral ‘fingerprint’, the identification of the elements constituting a given sample is, in principle, possible. In addition, under certain conditions, the quantitative analysis of a sample might also be possible. It is very important to note that the LIBS technique can be applied to all physical states of matter (i.e., solid, liquid or gaseous, metallic or dielectric), being capable of the simultaneous detection of multiple elements without any prior preparation of the sample and with good accuracy and fast response (e.g., within few milliseconds). All these characteristics have made LIBS very popular and an attractive technique for in situ elemental analysis applications [5,6,7,8,9].

The present study focuses on the investigation and the application of LIBS for the qualitative and quantitative determination of sulfur (S) in organic soil samples used in agriculture. Due to the lack of suitable spectral lines of sulfur for analytical use (i.e., qualitative and quantitative measurements) in the UV-Vis-NIR spectral region (200–1100 nm), the use of spectral lines lying in the vacuum ultraviolet (VUV) spectral region is examined and assessed here. To improve the sensitivity of the LIBS analysis, different statistical analysis methodologies are applied, such as uni- and multi-variate analyses, and are thoroughly assessed. To the best of our knowledge, this is the first time that some VUV spectral lines of sulfur are employed for analytical purposes, and a detailed investigation concerning the determination of sulfur content in organic soil matrices is carried out.

## 2. Materials and Methods

### 2.1. Experimental Setups

Two different experimental setups were constructed and used for the needs of the present investigation, namely one suitable for the spectral range from 175 to 200 nm (VUV), and the other operating in the 200–1200 nm (UV-Vis-NIR) spectral range. The former setup included a 200 mm focal length VUV monochromator (Mc Pherson 234 UHV) (Mc Pherson, Chelmsford, MA, USA), equipped with An MgF_2_ coated 1200 grooves/mm holographic grating, operating between 0.3 and 500 nm and having a dispersion of 3.37 nm/mm at 100 nm. The monochromator was equipped with an open front end CCD (1024 × 256 array, pixel size 26 μm) type detector (Andor DO420) (Andor Technology Oxford Instruments, Belfast, UK). The CCD detector was operating only in CW mode (i.e., without being gated) in the spectral region from 0.124 to 1200 nm. The second setup included a 75 mm focal length portable spectrograph (Avantes, Avaspec 2048) (Avantes BV, Apeldoorn, The Netherlands), with a 50 μm entrance slit, equipped with a CCD detector (2048 pixels, 0.44 nm/pixel).

**Figure 1:**
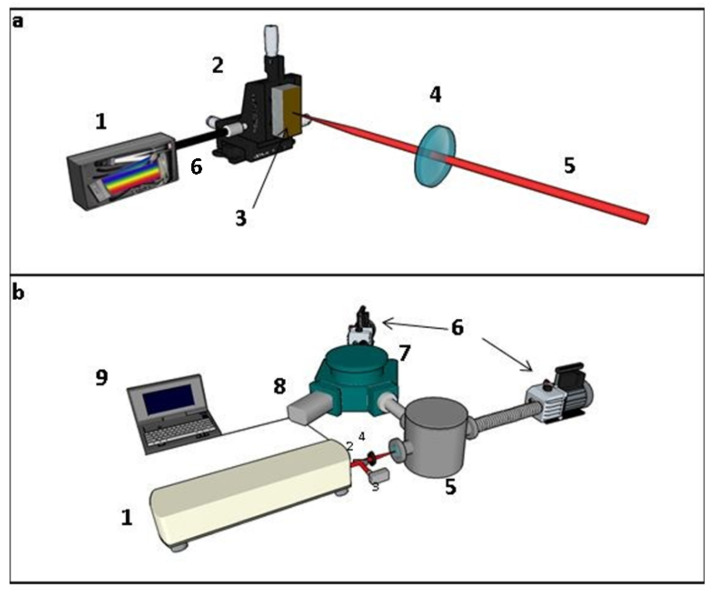
Experimental setups used: (**a**) 1: UV-VIS-NIR spectrograph, 2: XYZ translation stage, 3: sample, 4: focusing optics, 5: laser beam, 6: fiber bundle; (**b**) 1: Nd: YAG laser, 2: beam splitter 3: laser energy meter, 4: focusing optics, 5: vacuum chamber, 6: vacuum pump 7: vacuum ultraviolet (VUV) spectrograph, 8: CCD detector, 9: PC for data acquisition.

For both experimental setups, the samples were mounted on a XYZ translation stage, allowing the change in their position to avoid crater formation resulting from ten consecutive laser shots. For the VUV experiments, the sample was placed in a vacuum chamber and the laser beam was focused on the sample surface by means of a 400 mm plano-convex quartz lens. The pressure in the vacuum chamber was kept at 10^−2^ mbar, using a rotary vacuum pump. The light emitted from the plasma (produced in the vacuum chamber) was imaged on the entrance slit of the VUV monochromator, which was also maintained at the same pressure. The vacuum chamber was separated from the VUV monochromator by means of a lithium fluoride (LiF) window, ensuring the sealing between them. The exposure time of the CCD detector was set at 40 ms. For the experiments concerning the UV-Vis-NIR spectral region, the laser beam was focused on the sample’s surface by means of a 100 mm focal length quartz plano-convex lens. The light emitted from the plasma plume was collected in a direction perpendicular to the laser beam by a quartz lenses system and was introduced in a quartz fiber bundle which was coupled to the entrance slit of the spectrograph. For the CCD detector, the gating conditions used were a time delay (t_d_) of 1.28 μs and an integration time (t_w_) of 1.05 ms. Both experimental setups are shown schematically in Figure 1. 

In all cases, the plasma was created on the sample’s surface using the beam of a 5 ns Q-switched Nd:YAG laser (Spectra Physics, INDI) at 1064 nm, operating at a repetition rate of 1–10 Hz. The laser energy was measured by a calibrated joulemeter (Molectron J50-1073) (Molectron, Portland, OR, USA). Its fluctuation was less than 5%. To ensure a good signal-to-noise ratio while keeping the formation of craters on the samples’ surface as minimal as possible, the laser energy was set to about 100 mJ. The diameter of the beam at the focus was measured by a CCD and it was found to be about 38 μm for the 100 mm lens and 50 μm for the 400 mm lens. In all cases, the LIBS spectra from ten laser shots were averaged and used as one LIBS measurement. 

### 2.2. Samples Preparation 

**Figure 2:**
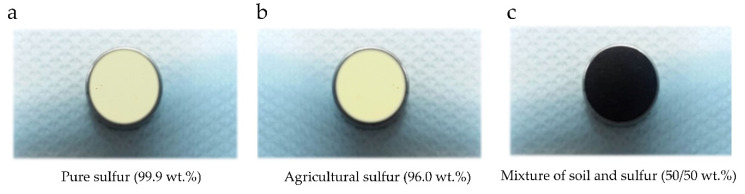
Samples prepared for the laser-induced breakdown spectroscopy (LIBS) measurements in the UV-ViS-NIR spectral region: (**a**) pure sulfur sample (99.9 wt.%), (**b**) agricultural sulfur (96.0 wt.%), (**c**) mixture of sulfur with organic soil (50/50 wt.%).

For the experiments concerning the UV-ViS-NIR spectral region, three types of sample were prepared. The first type consisted of pure sulfur (99.9 wt.%) (Figure 2a) and was used to determine the S emission lines observable under LIBS conditions, in the absence of any spectral interferences due to a soil matrix. For the second type of sample, commercially packaged sulfur (96 wt.%), usually employed for agricultural purposes, was used. In the next section, it will be referred as agricultural sulfur (Figure 2b). The comparison of the LIBS spectra of the pure S and agricultural sulfur samples allowed for the search for possible spectral interferences arising from the matrix. For the third type of sample, commercially packaged organic soil and agricultural sulfur (50/50 wt.%) were thoroughly mixed and used (Figure 2c). This last type of sample was used to search for the presence of any other interference effects on the S spectral lines arising from the organic soil matrix, and also to study the effect of the time delay, t_d_, on the LIBS spectra (as will be discussed in the next section). In all cases, the resulting samples were placed in a metallic ring and compressed to form a pellet, as shown in Figure 2.

For the part of the present study concerning the VUV spectral region, two sets of samples were prepared; both consisted of commercially packaged organic soil (containing 20% of various other unspecified organic substances according to the manufacturer), and agricultural sulfur. To increase the cohesivity of the samples and minimize crater formation under vacuum conditions, some starch powder was also added. From the two sets of samples, one was used for the construction of calibration curves, and the other for the validation of the accuracy of the calibration curves. Each set was comprised of eight different samples with different amounts of sulfur. The amounts of soil, sulfur and starch used for the preparation of the samples were weighed, mixed well with mortar and pestle, ground, and were compressed into pellets. The compositions of the prepared samples for the VUV measurements are presented in Table 1. All samples contained 10 g of organic soil, 0.4 g of starch and a variable amount of sulfur, ranging from 0 to 2.5 g, corresponding to a range of sulfur concentrations from 0 to 19.4 wt.%.

### 2.3. Data Statistical Analysis Methodologies

For the analysis of the experimental data, two statistical analysis methods were used, corresponding to two different types of calibration models, namely the univariate and multivariate methods. The former is known as the “calibration curves method” [10] and is a univariate analysis method used routinely for data analysis by several analytical techniques. It employs least squares regression (LSR) for the construction of a best fitting curve resulting from a set of known concentration (i.e., standard) samples (see, for example, Table 1). The term “univariate” denotes that only one variable (e.g., one specific wavelength corresponding to a spectral feature, or an integrated area, is used for the construction of the regression model. The latter method is a multivariate one and is called partial least squares regression (PLS-R). In this case, the term “multivariate” denotes that more than one variable (i.e., spectral feature), or even an entire LIBS spectrum, can be used as the input for the training of the regression model. According to that method, the original data are reduced to a smaller set of uncorrelated components (i.e., latent variables, LVs) and performs a linear regression to these components instead of the original data [11]. Additionally, the PLS-R algorithm can compute the statistical weight of each variable (e.g., wavelength/spectral feature), and these weights are called regression coefficients.

For the estimation of the accuracy of the regression models, the leave-one-out validation (LOOV) method was implemented. During this procedure, all the LIBS spectra, except one, are used as training data, while the remaining one is used for testing of the model. This procedure is repeated as many times as the number of LIBS spectra, and a score is obtained for every iteration. Then, the mean value of these scores is considered as the accuracy of the model. It must be pointed that for the estimation of the real performance of the regression models except for the LOOV method, an external validation was performed as well. For this reason, a validation set was constructed and used only for prediction purposes (see Samples preparation for more information).

For further evaluation of the regression models, the absolute error of the calibration curve method was calculated for the LSR model by comparing the concentration values from the output of the LSR model (predicted values) with the known concentration values [10,12]. On the other hand, the prediction error sum of squares (PRESS), which indicates whether the predictive ability of the model is sufficiently high, was evaluated and was used for the determination of the optimum number of latent variables. In more detail, the number of latent variables which corresponds to the minimum root mean PRESS value, was chosen for the algorithmic training [12,13,14]. 

Finally, the principal component analysis (PCA) algorithm was used for the estimation of the most suitable matrix to use as a binder in the samples. According to this method, the initial data are projected to a vector space, where the coordinate axes (i.e., the principal components, PCs) result from a linear combination of the initial data. In this way, the initial spectra can be represented as distinct points in the principal components vector space and, thus, multidimensional data (such as LIBS data) can be visualized in two or three dimensions for the inspection of possible pattern formation. The points that correspond with the spectra in the PC vector space are known as scores and the PCs are uncorrelated variables, which explain a specific amount of the original data variance. More specific, the first PC explains most of the original data variance, the second PC the next greatest amount of variance, and so on [15]. The above analyses were all performed using MATLAB software (R2020b, MathWorks, Natick, MA, USA).

## 3. Results

### 3.1. LIBS Investigations

In order to determine the sulfur spectral lines which can be observed in a soil organic matrix and to select the most suitable ones for quantification purposes, both the VUV (175–200 nm) and the UV-Vis-NIR (200–1200 nm) spectral regions were searched. Initially, the presence of sulfur lines previously reported in other studies was examined. Such studies referred to the elemental analysis of minerals [16], the detection and quantification of sulfur in petroleum derivatives [17,18], and the detection of sulfur in environmental samples [19,20]. In all these investigations, the sulfur spectral lines that were considered for analytical purposes lay in the UV and NIR spectral regions. In the present work, several LIBS experiments were performed on samples consisting of organic soil (purchased from the local market) enriched up to ~30% wt.% with sulfur and using different experimental conditions (e.g., laser energy, laser focusing conditions, etc.). In all cases and for all the experimental conditions used, the spectral lines of sulfur lying in the UV-ViS-NIR spectral region were found to be inadequate for quantitative analysis purposes, either because they were too weak to ensure a reasonable signal-to-noise ratio, or because, in most of the cases, they were found to overlap with spectral lines of other elements present in the organic soil matrix or in air. Interestingly, some sulfur emission lines were clearly observed as lying just below 200 nm. These lines were intense enough and relatively free from spectral interferences from other elements. So, they were selected to be used for qualitative and quantitative LIBS measurements.

**Figure 3:**
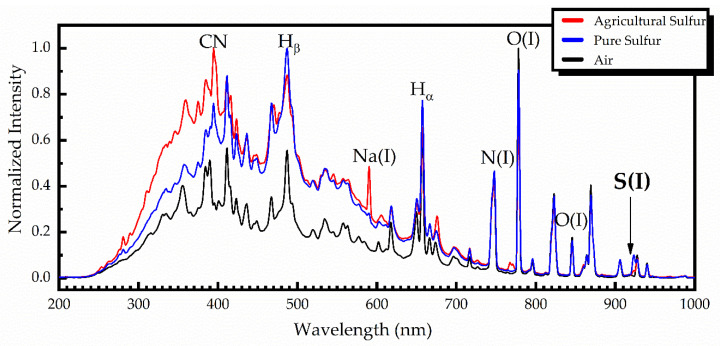
LIBS spectra of pure sulfur (blue), agricultural sulfur (red) and ambient air (black).

For comparison purposes, some characteristic LIBS spectra concerning the UV-ViS-NIR spectral region, obtained from pure and agricultural sulfur samples, are shown in Figure 3, together with a LIBS spectrum obtained in air. All these spectra were obtained using a time delay of 1.28 μs and an integration time of 1.05 ms. To facilitate comparison, the shown spectra are normalized.

**Figure 4:**
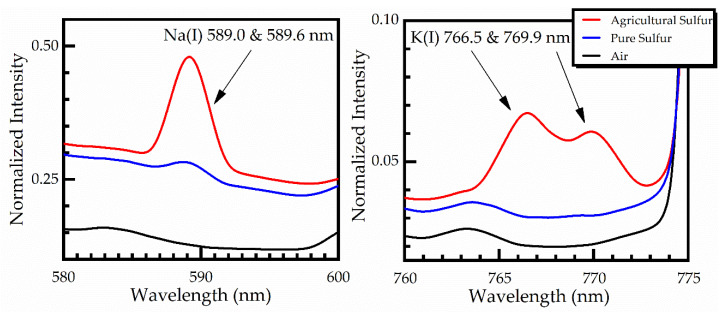
Sodium (Na) and Potassium (K) emission lines observed in the agricultural sulfur samples.

As can be seen from this figure, the LIBS spectra of pure and agricultural sulfur samples were very similar, exhibiting only minor differences, arising from the impurities contained in the agricultural sulfur samples. As an example, some of the observed differences are zoomed into and are shown in Figure 4. As shown, the sodium spectral lines Na(I) at 589.0 and 589.6 nm, and the potassium spectral lines K(I) at 766.5 and 769.9 nm are clearly observed in the agricultural sulfur LIBS spectra, while they are absent in the pure sulfur spectrum [21,22].

**Figure 5:**
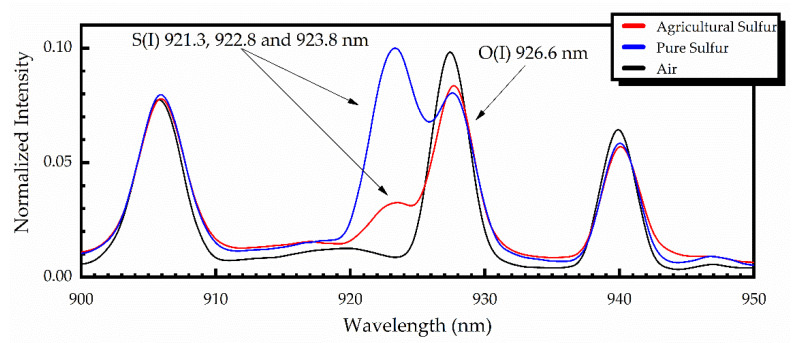
Unresolved emission lines of sulfur at 921.3, 922.8, and 923.8 nm, appearing as one line at about 923 nm, obtained from pure (blue) and agricultural (red) sulfur samples. The black line corresponds to the LIBS spectrum of air.

Similarly, in Figure 5, the LIBS spectra of pure and agricultural sulfur samples, together with the LIBS spectrum of air, in the NIR region between 900 and 950 nm are presented. In this spectral region, three spectral lines of sulfur are expected to appear, namely at 921.3 nm, 922.8 nm and 923.8 nm. However, due to the limited resolution of the spectrograph, the broadening of the lines, and their partial overlapping with the neighboring oxygen O(I) emission at 926.6 nm, they are not clearly observed. So, it was concluded that these NIR spectral lines of sulfur cannot be employed for quantitative purposes.

**Figure 6:**
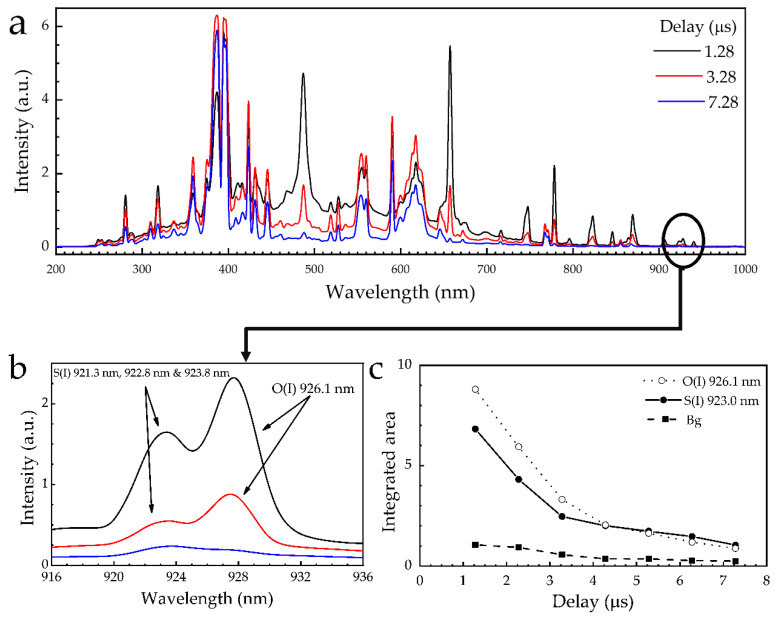
Effect of the time delay, t_d_, on: (**a**) the LIBS spectra of a soil-sulfur sample, (**b**) the sulfur spectral lines at around 923.0 nm (corresponding to a zoom of the black circle in (a)), (**c**) the peak intensities of the S(I) (black) and O(I) (blue) spectral lines and the corresponding background (red).

Next, the effect of the signal gating conditions on the LIBS spectra and more importantly on the strength of the sulfur spectral lines of the samples consisting from commercially packaged organic soil mixed with agricultural sulfur was studied. In the case of the UV-ViS-NIR measurements, the detector of the spectrograph could attain a minimum gate width (t_w_) and time delay (t_d_) of 1.05 ms and 1.28 μs, respectively. So, the effect of the time delay on the sulfur emissions was investigated for several t_d_ values, greater than 1.28 μs, keeping the integration time constant, i.e., at 1.05 ms. The obtained LIBS spectra are presented in Figure 6a. As it can be seen from the intensity dependence plots shown in Figure 6b,c, the spectral lines of S(I) and O(I) exhibit an exponential-like decay, decreasing rapidly within 2–3 μs. More precisely, for t_d_ ≥ 3.28 μs, both the S(I) and O(I) lines were observed to decrease significantly, resulting in a very low signal to noise ratio. This finding suggests that it is rather unfavorable to perform measurements for higher time delays. Based on this experimental evidence, and the relatively weak background observed (see also Figure 6c), it was concluded that the use of t_d_ lower than 3.28 μs was more appropriate for performing measurements. In addition, it is evident that the only suitable spectral line of sulfur for analytical use in these soil samples would be most probably the three S(I) lines at 921.3, 922.8, and 923.8 nm. However, these lines were found to remain weak under all experimental conditions tried.

Due to the aforementioned problems and limitations met while studying the LIBS spectra of the UV-VIS-NIR region, sulfur emissions in the near VUV spectral region were searched. Since in this case the samples were placed under vacuum conditions (i.e., in the vacuum chamber), where it was difficult to move them to avoid the problem of crater formation, the soil-sulfur samples were mixed with starch or lactose to improve their cohesivity. As a result, the crater formation was reduced significantly, improving the reproducibility of the measurements and greatly reducing the shot-to-shot variations of the plasma light intensity.

**Figure 7:**
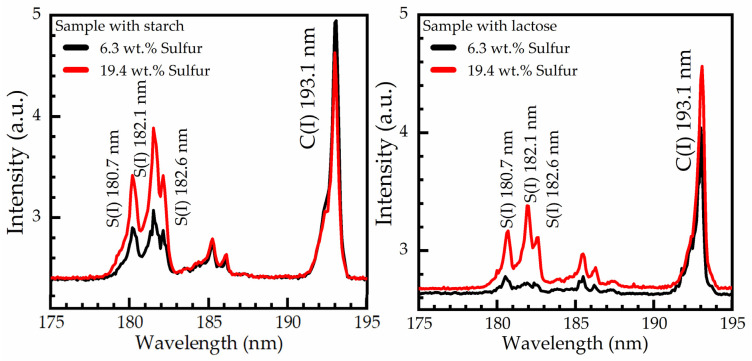
VUV emissions of LIBS spectra of soil-sulfur samples containing starch or lactose as binding material.

Some representative LIBS spectra of sulfur-soil samples with starch and lactose are illustrated in Figure 7. As can been seen in the LIBS spectra of Figure 7, three sulfur lines, namely the S(I) at 180.7 nm, S(I) at 182.1 nm, and S(I) at 182.6 nm, along with a carbon line C(I) at 193.1 nm, are clearly observed, while some weaker emissions at 185.6 and 186.3 nm, are tentatively attributed to different Fe (II) transitions. The spectroscopic information on the observed atomic transitions of interest was found from the NIST database [21] and is presented in Table 2.

In order to investigate possible contributions and/or other matrix effects arising from the addition of starch or lactose, LIBS measurements were performed on the samples containing starch and lactose under identical experimental conditions. As shown in Figure 7, the samples with starch as the binding material present higher intensities than the samples with lactose. In particular, in the case of lower sulfur concentration (see e.g., black lines), the emissions of S(I) from the lactose-sulfur sample almost vanished. These observations were confirmed by repeating the experiments using starch and lactose from different suppliers.

**Figure 8:**
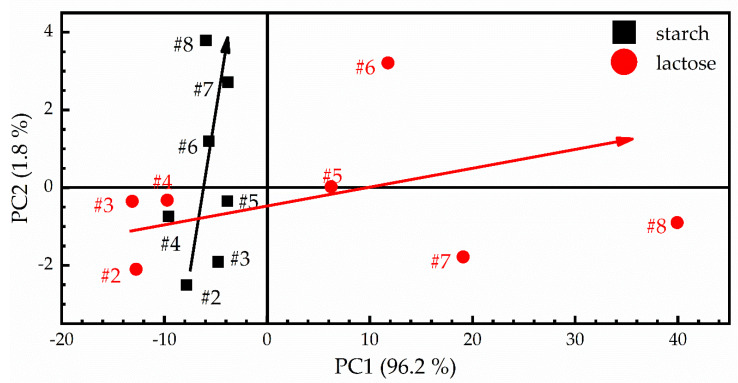
Scores diagram constructed from the LIBS spectra of the two samples’ sets containing starch and lactose. The numbers near the data points denote samples with different sulfur concentrations (see Table 1). The black and red arrows are indicating the samples’ distributions in the Principal Components plot.

To further explore the issue of the most suitable binder for the experiments, the PCA algorithm was implemented [22]. In Figure 8, the score plot of PCA for the mixed samples with starch and lactose, having identical concentrations with those shown of Table 1, is presented. As can be seen, two PCs explain approximately 98% (i.e., PC1 explains 96.2% and PC2 explains 1.8%) of the original data variance. The red dots correspond to the spectra of lactose-containing samples, while the black squares correspond to the starch-containing samples. From the score plot investigation, some patterns are clearly observable. At first, it seems that the starch-containing samples (black arrow) are mainly scattered at the PC2 axis, while the lactose-containing samples (red arrow) are scattered through the PC1 axes. In addition, both the lactose- and starch-containing samples exhibit the same behavior for low sulfur concentrations, since they have negative scores for both the PC1 and PC2 axes, while for a higher concentration, the lactose-containing samples have positive PC1 scores, in contrast with the starch-containing samples which have positive PC2 scores. Although the scores show an increasing behavior, it should be noted that, for lactose-containing samples, the dispersion of the scores becomes larger as sulfur’s concentration increases, while the scores of the starch-containing samples exhibit a more linear behavior and much smaller dispersion. These findings are strong evidence that the matrix effects in the presence of starch are much weaker than those related to the presence of lactose. As a result, we decided to use starch for increasing the cohesivity of the samples and to perform the following quantitative analyses.

### 3.2. Calibration Curves

Using the spectral information from the VUV spectral region, uni- and multi-variate calibration curves for sulfur were prepared.

#### 3.2.1. Univariate Analysis (Least Squares Regression (LSR))

The LSR algorithm was trained with the first set of samples for the construction of the calibration curve, while the second set of samples was used for the validation of the constructed model.

**Figure 9:**
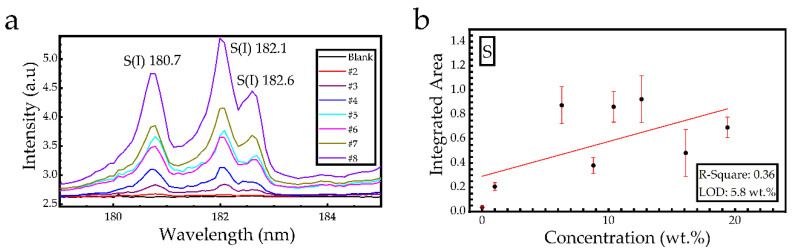
(**a**) LIBS spectra used for the calibration models. (**b**) Calibration curve obtained using linear regression.

**Figure 10:**
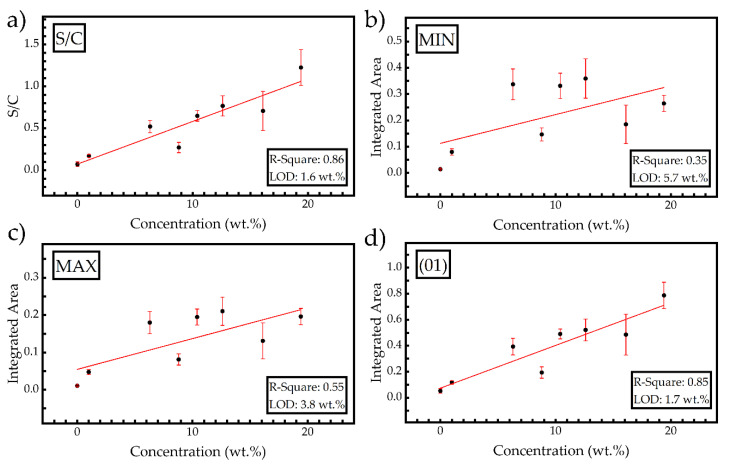
Calibration curves obtained using the different normalization methods, i.e., (**a**) S/C, (**b**) MIN, (**c**) MAX and (**d**) (01).

For the construction of the calibration curve, the area under the spectral lines located between 180 and 183 nm and presented in Figure 9a was used. The resulting calibration curve is presented in Figure 9b, and from the best fitting curve (red line), the resulting R-square value was estimated to be ~0.36, which is rather low for a predictive model. This non-successful curve fitting suggests that most likely, some underlying non-linear phenomena are present (e.g., matrix effects). In order to improve the results given by this method (hereafter denoted as “S” method), the data (spectra) were normalized by applying four different methods: by dividing the area below the S(I) lines with that of the C(I) spectral line (denoted as “S/C”), by dividing the area of S(I) lines with the minimum value of each spectrum (denoted as “MIN”), by dividing the area of the S(I) lines with the maximum value of each spectrum (denoted as “MAX”), and by normalizing all the spectra between a minimum and a maximum value, corresponding to 0 and 1, respectively (denoted as “(01)”). All calibration curves resulting from the above methods are presented in Figure 10. The red straight lines which are presented in this figure correspond to the best curve fitting to the experimental data. From slope b of each fitted line and the standard deviation σ_α_ of the background, the limits of detection (LOD) can be calculated using the expression [10,22]:

LOD (wt.%) = 3σ_α_/b

For the comparison of the different calibration curves obtained, both the R-square values and the corresponding limits of detection (LODs) were calculated. So, the R-square and LODs values, ranging from 0.35 to 0.86 and from 1.6 to 5.7 wt.%, respectively, were determined. In more detail, the calibration curve obtained from the S/C normalization method attained the best results and the lowest LODs, most probably because the samples contained a rather constant organic matter percentage, i.e., carbon, acting in this case as an internal standard [15], thus reducing the effect of matrix effects, laser pulse fluctuations, etc. Considering the MIN and MAX methods, it is obvious from the corresponding R-square values that the choice of the wing or peak intensity of a spectral line to normalize the LIBS spectra are not appropriate in the present case, as they result in a low R-square value, similar to that of the S calibration method (e.g., see Figure 9b). Finally, the use of the (01) normalization method resulted in a large R-square value, comparable to that of the S/C method. In fact, the (01) and the S/C normalization methods exhibited the most successful results, deviating about 10% from the ideal result, which is assumed to correspond to an R-square of 1 (or 100%). This deviation can be possibly attributed to the presence of some self-absorption, which should be expected as the present sulfur emissions arise either from resonant transitions (i.e., where the transition terminates at the ground state) or from transitions whose energy level is very low, as can be seen in Table 2. In this case, self-absorption can possibly and easily take place, causing considerable deterioration of the quality of the calibration curve and deviation from linearity. Some other issues may also explain the relatively large scattering of the data points, despite the careful preparation procedures followed to obtain well-mixed and homogeneous samples and sampling 10 laser shots for one LIBS spectrum. There are at least two factors that can affect the physical process of the laser-induced breakdown mechanism and, therefore, the spectral line emissions, i.e., the moisture content and the compression force used to prepare the pellets. The former has been reported to result in a reduction in the spectral line emissions, while the latter can result in increasing them, as more atoms can occupy the same volume of sample (see ref. [23] and references therein). The moisture content of the samples was very similar. However, the pressures used for the compression of the powdered sample were in a relatively large range (e.g., 50–100 tons/cm^2^) to avoid crater formation on the surface of the sample during the experiments taking place under vacuum conditions. 

After the evaluation of the different calibration curves using the first set of samples, the concentrations of the samples of the second set were predicted. As the concentrations of these samples are known, the absolute prediction errors were calculated for each of the previously described methods. In Table 3, the known concentrations (C_k_) are listed together with those predicted (C_p_) from the different calibration models. The corresponding absolute errors, defined as the absolute value of the difference between a predicted concentration and the known one, i.e., |C_p_ − C_k_|, are summarized in Table 4. As can be seen from Table 4, some of the calibration curves are more successful in predicting low concentrations of sulfur, while others seem to better predict higher concentrations. The most successful results were obtained via the S/C method (see, e.g., Figure 10a). Specifically, the absolute error of prediction for the 1 wt.% sulfur concentration is 0.2 wt.% (see Table 1) and was the lowest obtained compared to the rest of the normalization methods employed. The average absolute for this method, i.e., 2.5 wt.%, is the lowest obtained. The (01) method (see Figure 10d) also gave acceptable results, providing the best accuracy for the sample with a 6.3 wt.% concentration, corresponding to an absolute error of 0.5 wt.%. The average absolute error for this method was determined to be 2.9 wt.%. The other normalization methods resulted, in general, in slightly higher absolute errors, as can be seen in Table 4. 

There are several factors that can explain the less successful results of the S, MIN, MAX normalization methods. Among them, the most important are the possible presence of self-absorption [24] and matrix effects [23,25], which are unfortunately unavoidable. An extra problem arises from the formation of craters on the samples’ surface, even though starch powder has been used for limiting crater formation. Nevertheless, the S/C and (01) methods seem to significantly reduce the negative effects of these factors.

#### 3.2.2. Multivariate Analysis (Partial Least Square Regression Analysis (PLS-R))

**Figure 11:**
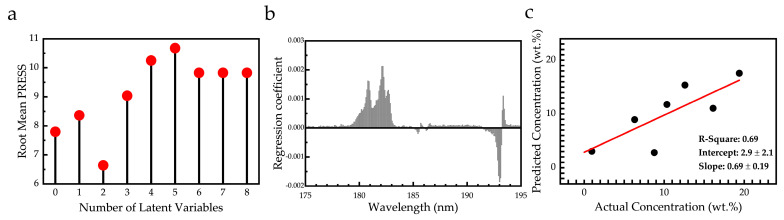
(**a**) Root mean prediction error sum of squares (PRESS) as a function of the number of the LVs. (**b**) Regression coefficients of each variable plotted versus wavelength. (**c**) Predicted versus actual value concentrations of the samples computed by partial least squares regression (PLS-R).

In the present work, the PLS-R calibration models were built using the LIBS spectra in the VUV region (i.e., 175 to 195 nm) as inputs (see, e.g., Figure 7 or Figure 9a). At first, the root mean PRESS values were computed for various numbers of LVs, in order to assess the optimum number of LVs for the training procedure. From this procedure, the plot in Figure 9a was obtained, and the optimum number of LVs was found to be 2, since this value corresponds to the minimum root mean PRESS value. Then, the regression coefficients were computed for each input variable (e.g., wavelength) and they were plotted against the wavelengths (see Figure 11b). In that way, the spectral data which contributed the most to the training of the algorithm could be determined. As can be seen, the spectral variables that are used in the PLS-R model correspond to the sulfur S(I) lines and the carbon line C(I), while the iron lines Fe(II) seem to not affect the model. Finally, the PLS-R model was trained with the first set of eight samples and evaluated by predicting the second set of the eight known concentration samples. From this procedure, a plot comparing the actual and predicted concentrations of the samples was constructed, and the R-square value was computed. This plot is presented in Figure 11c and the corresponding R-square value was determined to be 0.69.

For further improvement of PLS-R model performance, different normalization methods were performed on the input spectra and the resulting models were evaluated. Similarly with the normalization procedures employed for the univariate analysis, the spectra were normalized, by division with the C(I) line (S/C), with either the minimum or maximum value of each spectrum (MIN and MAX, respectively) and by scaling each spectrum from 0 to 1 (01). The optimum number of LVs, root mean PRESS and R-square values for each one of the abovementioned cases are shown in Table 5. The best results were obtained by using the normalization methods “S/C” and “(01)”.

Then, the constructed PLS-R models were used to predict the concentrations of the second set of samples. The actual and the predicted values are summarized in Table 6, while the absolute error of each method is given in Table 7. As can be seen from the former table, in all cases, the mean absolute error is low and, comparable to the univariate analysis results, and moreover, the PLS-R models seem to have a better performance. In more detail, minimum mean absolute error of the PLS-R method was calculated to be of ~2 wt.% for both the S/C and (01) methods, which are quite close to the values attained from the LSR models.

From the obtained results, it becomes evident that PLS-R analysis can improve the predictions regarding the analysis using LSR. This occurs because PLS-R takes into account variables from the whole spectral range and the influence of various spectral features on the final results can be accounted for. Moreover, the comparison of the results of the two statistical analysis methods, i.e., the univariate (LSR) and the multivariate (PLS-R) ones, indicate that the effects of the various complex physicochemical processes occurring during laser–matter interactions can be significantly reduced. The aforementioned results strengthen the analytical capabilities of the LIBS technique when it is combined with such statistical analysis.

## 4. Conclusions

In the present work, the LIBS technique was used to provide qualitative and quantitative information about the detection and determination of sulfur content in organic soil. For this purpose, both the VUV and UV-VIS-NIR spectral regions were studied in order to choose the most suitable sulfur spectral lines which can be used. It was found that sulfur lines in the UV-VIS-NIR spectral region are either very weak and/or significantly quenched due to matrix effects. On the contrary, some spectral lines lying in the VUV spectral region have been found to be less affected by matrix effects and, for this reason, these emissions have been selected for quantitative measurements by constructing calibration curves. To improve the performance of the calibration curves different statistical methods were employed, such as univariate (LSR) and multivariate (PLS-R) approaches. In addition, different types of normalization of the spectroscopic data were performed, allowing the minimization of effects, such as various matrix effects, laser energy fluctuations, stochasticity of the plasma production and evolution, etc. Finally, the accuracy of the detection of sulfur concentration in soil has been greatly improved. Among the methods tested, the PLS-R technique was found to be more efficient that the LSR technique, since it can use a wide range of spectral information as input and seems to be less affected by matrix effects, etc.

## Figures and Tables

**Table 1:** Composition of the soil samples used for LIBS measurements in the VUV spectral region.

Sample	Soil (g)	Sulfur (g)	Starch (g)	Concentration (wt.%)
#1	10.0	-	0.4	0
#2	10.0	0.1	0.4	1.0
#3	10.0	0.7	0.4	6.3
#4	10.0	1.0	0.4	8.8
#5	10.0	1.2	0.4	10.4
#6	10.0	1.5	0.4	12.6
#7	10.0	2.0	0.4	16.1
#8	10.0	2.5	0.4	19.4

**Table 2:** Characteristics (i.e., wavelength, transition probability (A_ki_) and lower and upper energy levels (E_i_, E_k_)) of the S(I) and C(I) spectral lines observed in the spectral window 180–200 nm.

Element	Wavelength (nm)	A_ki_ (10^8^ s^−1^)	E_i_ (eV)	E_k_ (eV)
S(I)	180.7	3.27	0	6.86
S(I)	182.1	1.71	0.04	6.86
S(I)	182.6	0.56	0.07	6.86
C(I)	193.1	3.39	1.26	7.68

**Table 3:** Comparison of the known concentrations (C_k_) with the predicted ones (C_p_).

Known Concentration—C_k_ (wt.%)	Predicted Concentration—C_p_ (wt.%)				
-	Method				
-	S	S/C	MIN	MAX	(01)
1	−0.3	1.2	−0.9	−1.3	0.3
6.3	18.4	7.5	18.5	11.7	6.8
8.8	9.7	7.5	9.5	7.9	6.7
10.4	40.8	16.5	41.5	29.1	17.7
12.6	16.1	14.7	15.6	15.7	14.4
16.1	63.1	21.7	64.0	43.4	23.5
19.4	26.7	18.8	26.9	23.9	20.0

**Table 4:** Absolute error (|C_p_ − C_k_|) occurring in each calibration method (wt.%).

S	S/C	MIN	MAX	(01)
1.3	0.2	1.9	2.3	0.7
12.1	1.2	12.2	5.4	0.5
0.9	1.3	0.7	0.9	2.1
30.4	6.1	31.1	18.7	7.3
3.5	2.1	3.0	3.1	1.8
47	5.6	47.9	27.3	7.4
7.3	0.6	7.5	4.5	0.6
**Average absolute error (%)**				
14.7	2.5	14.9	8.9	2.9

**Table 5:** Characteristic values derived from the PLS-R model.

Method	LVs	Root Mean PRESS (wt.%)	R-Square
S	2	6.7	0.69
S/C	1	4.0	0.79
MIN	2	6.7	0.71
MAX	2	7.2	0.64
(01)	2	5.0	0.81

**Table 6:** Actual and predicted values of the different calibration methods.

Actual Value (wt.%)	Predicted Value (wt.%)				
-	Method				
-	S	S/C	MIN	MAX	(01)
1	−4.0	2.8	−4.2	−0.7	3.1
6.3	8.9	8.0	8.5	7.8	8.0
8.8	11.0	10.0	10.4	10.0	10.0
10.4	18.1	13.5	17.2	16.0	13.3
12.6	15.1	14.2	15.0	14.6	13.9
16.1	17.1	18.6	25.1	23.4	18.6
19.4	17.5	17.3	17.4	18.0	17.0

**Table 7:** Absolute errors of each method.

S	S/C	MIN	MAX	(01)
5.0	1.8	5.2	1.7	2.1
2.6	1.7	2.2	1.5	1.7
2.2	1.2	1.6	1.2	1.2
7.7	3.1	6.8	5.6	2.9
2.5	1.6	2.4	2.0	1.3
1.0	2.5	9.0	7.3	2.5
1.9	2.1	2.0	1.4	2.4
**Average absolute error (%)**				
3.3	2.0	4.2	3.0	2.0

## Data Availability

Data sharing not applicable.
